# Comparison of Postoperative Muscle Strength Recovery Between Cisatracurium and Rocuronium

**DOI:** 10.7759/cureus.66767

**Published:** 2024-08-13

**Authors:** Vidya Rani M, Afra Farheen Faiaz, Shanthala K Banakara, Jyothsna Gopinathan NK, Mohammed Inayathulla Khan, Sunil H C, Ravi Kumar Yeli

**Affiliations:** 1 Department of Anesthesiology, Kanachur Institute of Medical Sciences, Mangalore, IND; 2 Department of Anesthesiology, Patil Ortho and Trauma Centre, Ilkal, IND; 3 Department of Orthopedics, Yenepoya Medical College and Hospital, Mangalore, IND; 4 Department of Radiology, Kanachur Institute of Medical Sciences, Mangalore, IND; 5 Department of Radiology, Shri B M Patil Medical College Hospital and Research Centre, Bijapur Lingayat District Educational University, Vijayapura, IND

**Keywords:** curarization, muscle strength, cisatracurium, rocuronium, anesthesia, neuromuscular blocking agents

## Abstract

Background

Neuromuscular blocking agents are crucial for anesthesia but can cause reversible paralysis, leading to risks like postoperative residual dysfunction. Undetected paralysis in the post-anesthesia care unit (PACU) jeopardizes patient safety by impairing airway function and increasing complications. Effective reversal, assessed clinically or via nerve stimulation, is critical to prevent residual postoperative curarization (RPOC), which is linked to significant morbidity and mortality. Evaluating agents like rocuronium and cisatracurium helps optimize anesthesia outcomes and patient recovery.

Methodology

The study included 100 American Society of Anaesthesiologists (ASA) I and II patients approved by the Institutional Review Board of Kasturba Medical College, Mangalore, India. Patients were briefed about the study, provided written informed consent, and underwent pre-anesthetic evaluations, including discussions on anesthetic procedures and associated risks. They were instructed to fast overnight after consenting.

Results

The study compared 100 ASA I and II patients receiving rocuronium or cisatracurium during anesthesia, analyzing age distribution (p=0.429), gender (p=0.839), ASA status (p=0.228), and physical characteristics (height, weight, BMI, p>0.05). Recovery parameters such as hand grip, sustained head lift, and double burst stimulation (DBS) twitch response showed no significant differences between groups (p=0.538 for hand grip and sustained head lift; p=0.220 for DBS. Late recovery rates at 15 minutes were observed with 16% for hand grip, 14% for sustained head lift, and 26% for DBS in the rocuronium group; compared to 14%, 10%, and 16%, respectively, in the cisatracurium group.

Conclusion

The study found significant postoperative residual curarization in both groups, emphasizing the need for intraoperative and PACU peripheral nerve stimulation for effective assessment. Further research on intraoperative variables could improve understanding of residual paralysis in PACU, guiding better anesthesia management.

## Introduction

Neuromuscular blocking agents are broadly used during the induction and maintenance of anesthesia. They facilitate intubation and ease the surgery. However, they cause reversible paralysis, which might sometimes linger at the end of surgical and anesthetic procedures. For safe and successful outcomes of surgery, it is important to understand the appropriate control of the quality and duration of the neuromuscular blockade (NMB). It also becomes critical to ensure the complete reversal of and recovery from the NMB before the patient is handed over to the post-anesthesia care unit (PACU).

Undetected residual paralysis of neuromuscular function in the PACU can jeopardize patients due to upper airway muscle weakness, increase the risk of pulmonary complications by reducing the ventilatory responses to hypoxemia, and increase the risk of aspiration [1], upper airway obstruction [2], visual trouble, and dysphagia [1,2]. It can also worsen postoperative well-being, increase the length of PACU stay, and ultimately necessitate reintubation [3]. Hence, the monitored use of and recovery from neuromuscular blockers is important.

Complete reversal of an NMB can be assessed clinically or objectively using a nerve stimulator after administering the reversal agent. Adequate reversal eliminates the possibility of residual paralysis and therefore prevents the associated complications. Postoperative residual muscle paralysis, or residual postoperative curarization (RPOC), occurs due to the incomplete antagonism of non-depolarizing muscle relaxants. RPOC is associated with major morbidity and mortality. Complete reversal can be confirmed using a nerve stimulator after administering the reversal agent or through clinical evidence of recovery, ensuring that complications related to residual paralysis are avoided.

In some European countries, such as Germany and Belgium, cholinesterase inhibitors are used for the reversal of NMBs in only 25% of cases, making it not the first-line approach worldwide [4,5]. Meticulous monitoring and thorough titration of the muscle relaxant can help avoid overdosing patients with NMBs.

Rocuronium bromide is a steroid-based, mono-quaternary amine with a rapid onset and intermediate-acting, relatively low-potency neuromuscular blocking drug. Due to its lipophilic nature, the majority of the drug is primarily taken up by the liver and eliminated via the biliary route [6]. Cisatracurium belongs to the group of benzylisoquinoline compounds and is a cis-isomer of atracurium. About 77% of the drug is metabolized by Hoffman's elimination to laudanosine, and 15% is excreted unchanged in urine. It is four times more potent than atracurium and has a moderately longer onset and duration of action [6].

The aim of this study was to determine the incidence of postoperative residual curarization between cisatracurium and rocuronium, providing insight into their respective efficacies in ensuring complete recovery from NMBs.

## Materials and methods

Study design and setting

The study was designed as an observational study and conducted in Kasturba Medical College (KMC) Hospitals, Mangalore, India. Following the approval of the Institutional Ethics Committee, the study was carried out. The study population included patients admitted for surgeries under general anesthesia.

Selection Criteria

The study included patients who underwent procedures under general anesthesia lasting at least 60 minutes with the need to use neuromuscular-blocking agents. Participants were aged between 18 and 80 years and classified as American Society of Anaesthesiologists (ASA) classes 1 and 2. Additionally, patients had to be awakened and extubated in the operating room (OR) and continue to be monitored in the PACU for at least 60 minutes. Subjects who were unable to give valid consent were excluded from the study. Other exclusion criteria included patients in emergency situations, those not awakened or not extubated in the OR, individuals above 80 years or under 18 years of age, surgical operations lasting less than an hour, and patients classified as ASA physical health classes 3 and 4. Additionally, patients with an impossibility to apply or perform neuromuscular monitoring in the PACU, those with neuromuscular diseases, individuals taking other drugs interfering with neuromuscular function, and pregnant or breastfeeding women were excluded from the study.

Sample Size Determination and Study Parameters

The sample size was determined based on prior studies examining the effects of neuromuscular-blocking agents under general anesthesia, with a power analysis estimating the need for 100 patients to detect a clinically significant difference with 80% power and a 5% significance level [4]. The upper age limit of 80 years was selected due to the increased risk of complications and altered pharmacodynamics in elderly patients, as they often have multiple comorbidities and a higher ASA classification, which could confound results. To ensure patient safety and maintain homogeneity, the upper age limit was set at 80 years. The study focused on procedures lasting at least 60 minutes; however, for practical purposes, the upper limit of surgery duration was set at 240 minutes (four hours) to encompass the typical duration of surgeries involving neuromuscular-blocking agents and to allow a comprehensive assessment of these agents' effects.

Data sources and variables

The study included 100 ASA I and II patients, approved by the Institutional Review Board of KMC, Mangalore. Patients meeting the inclusion criteria were briefed about the study, and written informed consent was obtained. None dropped out or were disqualified. Pre-anesthetic check-ups were conducted, explaining the anesthetic technique, induction procedure, benefits, risks, delayed reversal, and residual paralysis management. After consent, patients were advised to fast overnight.

Patients were randomized using a sealed envelope method into two groups: group ROC received rocuronium 1.2 mg/kg IV, and group CIS received cisatracurium 0.2 mg/kg IV. Standard anesthetic techniques were used after securing an IV line, and baseline vital parameters were recorded. Both groups were induced with fentanyl 2 mcg/kg IV and propofol 2 mg/kg IV in quantified boluses. Intubation was performed after induction (60 seconds for ROC and 90 seconds for CIS), and the maintenance phase was conducted as usual. Patients were extubated after clinical recovery and reversal of the block with neostigmine 0.05 mg/kg IV.

The procedure involved a questionnaire after clinical assessment, the conduct of anesthesia, admission to the PACU, and measurement of fade on double burst stimulation (DBS) using a neuromuscular monitor (NSML 100, Inmed Equipments Pvt. Ltd., Vadodara, India). This was done upon arrival in the PACU and repeated every five minutes up to the 15th minute. Patients were monitored for clinical recovery and DBS at 10, 15, 30, and 60 minutes after extubation. The persistence of the block, defined as the presence of fade, was assessed, and such patients were closely monitored and managed supportively as needed.

Statistical analysis

Statistical data were analyzed using IBM SPSS Statistics for Windows, Version 17, (Released 2008; IBM Corp., Armonk, New York, United States). The qualitative data were assessed with the student unpaired t-test and the chi-square test, as applicable. A p-value of less than 0.05 was considered statistically significant. A printed proforma was used to collect the data, which was then securely tabulated as a Microsoft Excel sheet (Microsoft Corporation, Redmond, Washington, United States).

## Results

Table 1 shows the comparison of different age groups in the study. In the age group 20-30, there were seven patients (14.0%) in the ROC group and 11 patients (22.0%) in the CIS group, making a total of 18 patients (18.0%). In the age group 31-40, there were 13 patients (26.0%) in the ROC group and six patients (12.0%) in the CIS group, with a total of 19 patients (19.0%). In the age group 41-50, both groups had 14 patients (28.0%), making a total of 28 patients (28.0%). In the age group 51-60, there were 11 patients (22.0%) in the ROC group and 14 patients (28.0%) in the CIS group, totaling 25 patients (25.0%). For patients above 60, each group had five patients (10.0%), making a total of 10 patients (10.0%). Overall, there were 50 patients in each group, making a total of 100 patients. The chi-square test p-value was 0.429, indicating no significant difference.

**Table 1:** Comparison of different age groups in the study Chi-square test p=0.429, not significant

			Group		Total
			Rocuronium	Cisatracurium	
Age	20 – 30	Count	7	11	18
		% within group	14.0%	22.0%	18.0%
	31 – 40	Count	13	6	19
		% within group	26.0%	12.0%	19.0%
	41 – 50	Count	14	14	28
		% within group	28.0%	28.0%	28.0%
	51 – 60	Count	11	14	25
		% within group	22.0%	28.0%	25.0%
	Above 60	Count	5	5	10
		% within group	10.0%	10.0%	10.0%
Total		Count	50	50	100
		% within group	100.0%	100.0%	100.0%

Table 2 shows the comparison of gender between the groups. In the ROC group, there were 22 females (44.0%) and 28 males (56.0%). In the CIS group, there were 21 females (42.0%) and 29 males (58.0%). Overall, there were 43 females (43.0%) and 57 males (57.0%), with 50 patients in each group and a total of 100 patients. The chi-square test p-value was 0.839, indicating no significant difference.

**Table 2:** Comparison of gender between the groups Chi-square test p=0.839, not significant

			Group		Total
			Rocuronium	Cisatracurium	
Sex	Female	Count	22	21	43
		% within group	44.0%	42.0%	43.0%
	Male	Count	28	29	57
		% within group	56.0%	58.0%	57.0%
Total		Count	50	50	100
		% within group	100.0%	100.0%	100.0%

Table 3 shows the comparison of ASA status between the groups. In the ROC group, 30 patients (60.0%) were classified as ASA 1 and 20 patients (40.0%) as ASA 2. In the CIS group, 24 patients (48.0%) were ASA 1 and 26 patients (52.0%) were ASA 2. Overall, there were 54 patients (54.0%) classified as ASA 1 and 46 patients (46.0%) as ASA 2, with 50 patients in each group and a total of 100 patients. The chi-square test p-value was 0.228, indicating no significant difference.

**Table 3:** Comparison of ASA status between the groups ASA: American Society of Anesthesiologists Chi-square test p = 0.228, not significant

			Group		Total
			Rocuronium	Cisatracurium	
ASA	1.	Count	30	24	54
		% within group	60.0%	48.0%	54.0%
	2.	Count	20	26	46
		% within group	40.0%	52.0%	46.0%
Total		Count	50	50	100
		% within group	100.0%	100.0%	100.0%

Table 4 shows the comparison of height, weight, and BMI between the groups. The mean height for the ROC group was 52.38 with a standard deviation of 3.72, and for the CIS group, it was 52.00 with a standard deviation of 2.47. The total mean height was 52.19 with a standard deviation of 3.15. The t-test p-value for height was 0.549, indicating no significant difference. The mean weight for the ROC group was 154.78 with a standard deviation of 4.08, and for the Cisatracurium group, it was 154.54 with a standard deviation of 3.01. The total mean weight was 154.66 with a standard deviation of 3.57. The t-test p-value for weight was 0.739, indicating no significant difference. The mean BMI for the ROC group was 20.98 with a standard deviation of 1.02, and for the CIS group, it was 20.66 with a standard deviation of 1.02. The total mean BMI was 20.82 with a standard deviation of 1.03. The t-test p-value for BMI was 0.120, indicating no significant difference.

**Table 4:** Comparison of height, weight, and BMI between the groups

		N	Mean	Standard deviation	t-test p-value	
Height	Rocuronium	50	52.38	3.72	0.549	Not significant
	Cisatracurium	50	52.00	2.47		
	Total	100	52.19	3.15		
Weight	Rocuronium	50	154.78	4.08	0.739	Not significant
	Cisatracurium	50	154.54	3.01		
	Total	100	154.66	3.57		
BMI	Rocuronium	50	20.98	1.02	0.120	Not significant
	Cisatracurium	50	20.66	1.02		
	Total	100	20.82	1.03		

Table 5 shows the comparison of hand grip between the groups. In the ROC group, 42 patients (84.0%) had a hand grip duration of less than 10 minutes, and eight patients (16.0%) had a hand grip duration of less than 15 minutes. In the CIS group, 47 patients (94.0%) had a hand grip duration of less than 10 minutes, and three patients (6.0%) had a hand grip duration of less than 15 minutes. Overall, 89 patients (89.0%) had a hand grip duration of less than 10 minutes, and 11 patients (11.0%) had a hand grip duration of less than 15 minutes. The chi-square test p-value was 0.538, indicating no significant difference.

**Table 5:** Comparison of hand grip between the groups Chi-square test p = 0.538, not significant

		Group				Total	
		Rocuronium		Cisatracurium			
		Count	%	Count	%	Count	%
Hand grip	<10 min	42	84.0%	47	94.0%	89	89.0%
	<15 min	8	16.0%	3	6.0%	11	11.0%
Total		50	100.0%	50	100.0%	100	100.0%

Table 6 shows the comparison of sustained head lift between the groups. In the ROC group, 43 patients (86.0%) had a sustained head lift duration of less than 10 minutes, and seven patients (14.0%) had a duration of less than 15 minutes. In the CIS group, 45 patients (90.0%) had a sustained head lift duration of less than 10 minutes, and five patients (10.0%) had a duration of less than 15 minutes. Overall, 88 patients (88.0%) had a sustained head lift duration of less than 10 minutes, and 12 patients (12.0%) had a duration of less than 15 minutes. The chi-square test p-value was 0.538, indicating no significant difference.

**Table 6:** Comparison of sustained head lift between the groups Chi-square test p = 0.538, not significant

		Group				Total	
		Rocuronium		Cisatracurium			
		Count	%	Count	%	Count	%
Sustained head lift	<10 min	43	86.0%	45	90.0%	88	88.0%
	<15 min	7	14.0%	5	10.0%	12	12.0%
Total		50	100.0%	50	100.0%	100	100.0%

Table 7 shows the comparison of twitch response for double burst stimulation between the groups. In the ROC group, 37 patients (74.0%) had a twitch response duration of less than 10 minutes, and 13 patients (26.0%) had a duration of less than 15 minutes. In the CIS group, 42 patients (84.0%) had a twitch response duration of less than 10 minutes, and eight patients (16.0%) had a duration of less than 15 minutes. Overall, 79 patients (79.0%) had a twitch response duration of less than 10 minutes, and 21 patients (21.0%) had a duration of less than 15 minutes. The chi-square test p-value was 0.220, indicating no significant difference.

**Table 7:** Comparison of twitch response for double burst stimulation (DBS) between the groups Chi-square test p = 0.220, not significant

		Group				Total	
		Rocuronium		Cisatracurium			
		Count	%	Count	%	Count	%
DBS	<10 min	37	74.0%	42	84.0%	79	79.0%
	<15 min	13	26.0%	8	16.0%	21	21.0%
Total		50	100.0%	50	100.0%	100	100.0%

Table 8 shows the average number of patients who recovered late in both groups. In the ROC group, eight patients (16.0%) had a hand grip duration of 15 minutes, seven patients (14.0%) had a sustained head lift duration of less than five seconds, and 13 patients (26.0%) had a DBS duration of 15 minutes. In the CIS group, seven patients (14.0%) had a hand grip duration of 15 minutes, five patients (10.0%) had a sustained head lift duration of less than five seconds, and eight patients (16.0%) had a DBS duration of 15 minutes. Overall, six patients (11.0%) had a hand grip duration of 15 minutes, 12 patients (12.0%) had a sustained head lift duration of less than five seconds, and 21 patients (21.0%) had a DBS duration of 15 minutes.

**Table 8:** Average number of patients who recovered late in the two groups DBS: double burst stimulation

Time: 15 minutes	Group				Total	
	Rocuronium		Cisatracurium			
	Count	%	Count	%	Count	%
Hand grip	8	16%	7	14%	6	11.0%
Sustained head lift < five seconds	7	14%	5	10%	12	12.0%
DBS	13	26%	8	16%	21	21.0%

## Discussion

Postoperative morbidity can occur in patients recovering from anesthesia, where a depressed neuromuscular response due to residual paresis continues beyond the anesthesia recovery period. Despite the widespread use of intermediate-acting NMBs, postoperative residual curarization is still frequent in the PACU [7-11]. In the present study, when the recovery index was measured using DBS in a total of 100 patients, 21 patients had a delayed recovery at 15 minutes in which group ROC had 13 (26%) patients and group CIS had eight (16%). Although the CIS group had a lesser incidence compared to the ROC group, the difference was statistically insignificant.

Xiaobo et al. [10] compared the recovery index between rocuronium and cisatracurium, comparing adults and the elderly. They found recovery from rocuronium took 17 min in adults and 22 min in the elderly group whereas cisatracurium showed 15.3+25 minutes in adults and 15.5+2.2 minutes in the elderly. The average time for the recovery index emerged to be remarkably longer and variability was much greater for the rocuronium group, compared to the cisatracurium group. Although clinical assessment has a lower sensitivity compared to qualitative assessment, combining both will help us detect and reduce residual paralysis. In our study, on comparing the hand grip in 100 patients, 11% of patients had a recovery at 15±8 minutes measured from the time of shifting to PACU. Group ROC had eight (16%) patients, group CIS had three (6%) patients recovering at 15 minutes from the time of entering the PACU (chi-square test p-value = 0.110). Results of sustained head lift > five seconds showed that out of 100 patients, 12 had a recovery time of 15 minutes, out of which group ROC had seven (14%) and group CIS had five (10%) patients who had a later recovery of 15 minutes from the time of arrival to PICU (chi-square test p-value = 0.538).

In a study conducted by Invernizzi et al. [12], using a train-of-four (TOF) ratio for assessment of residual paralysis, they demonstrated a higher incidence of TOF ratio of <0.9 in patients who received rocuronium compared to cisatracurium, although the results were statistically insignificant. Viby-Mogensen et al. [13] used TOF nerve stimulation during anesthesia as well as postoperatively for titration of reversal with anticholinesterase drugs. Cammu et al. [14] observed no notable variability in recovery indices between cisatracurium and rocuronium. The TOF ratios at 10 minutes post-reversal were 0.7+0.1 and 0.7+0.1, respectively. At 15 minutes post-reversal, one patient had a TOF ratio <0.7 in each individual group. There were no patients in either group with a TOF ratio < 0.7 on shifting to the recovery unit.

On relating the above studies with our results, there is a conflicting opinion between both the drugs and their variability in recovery characteristics, where in some studies, cisatracurium had statistically significant better recovery outcomes compared to rocuronium, but in ours, even though cisatracurium had a better recovery profile compared to rocuronium, the results were not statistically significant. This slight difference of opinion in the study by Maybauer et al. [15] can be explained by the outcome of blood loss and propofol on the metabolism of both the drugs: cisatracurium being metabolized independently of liver and renal function via Hoffman's elimination (pH- and temperature-dependent). In the clearance of rocuronium, hepatic elimination acts as a major pathway. Propofol, as well as major surgery or abdominal surgery in patients with significant blood loss, significantly decreases hepatic blood flow. This increases NMB effects. Therefore, there is a speculated difference between cisatracurium and rocuronium in the time of action and variability in recovery characteristics as recognized in various studies.

Limitations of the study

The study had several limitations that should be noted. Firstly, a larger sample size would have been necessary to achieve greater statistical power and potentially detect smaller, clinically significant differences between the groups. Secondly, the addition of TOF alongside DBS could have provided additional confirmation and comparison of safe recovery from NMBs. Thirdly, while the null hypothesis was confirmed, it would have been beneficial to rule out any unintended confounding factors, such as variations in environmental temperature. Precautions were taken to minimize sampling and experimental errors throughout the study.

## Conclusions

The study revealed a notable incidence of postoperative residual curarization in both groups, underscoring the critical role of peripheral nerve stimulation for assessing NMB both intra-operatively and in the PACU. The strategic administration of muscle relaxants and the systematic evaluation of residual paralysis are pivotal in mitigating associated morbidity and complications linked to residual curarization. Expanding the study to include intraoperative variables could provide deeper insights into the factors contributing to persistent NMB during the recovery phase in the PACU. Such investigations are crucial for refining clinical practices and optimizing patient outcomes in anesthesia management.

## References

[1] Sundman E, Witt H, Olsson R, Ekberg O, Kuylenstierna R, Eriksson LI (2000). The incidence and mechanisms of pharyngeal and upper esophageal dysfunction in partially paralyzed humans: pharyngeal videoradiography and simultaneous manometry after atracurium. Anesthesiology.

[2] Eikermann M, Groeben H, Hüsing J, Peters J (2003). Accelerometry of adductor pollicis muscle predicts recovery of respiratory function from neuromuscular blockade. Anesthesiology.

[3] Berg H, Roed J, Viby-Mogensen J, Mortensen CR, Engbaek J, Skovgaard LT, Krintel JJ (1997). Residual neuromuscular block is a risk factor for postoperative pulmonary complications. A prospective, randomised, and blinded study of postoperative pulmonary complications after atracurium, vecuronium and pancuronium. Acta Anaesthesiol Scand.

[4] Cammu G, De Witte J, De Veylder J (2006). Postoperative residual paralysis in outpatients versus inpatients. Anesth Analg.

[5] Fuchs-Buder T, Hofmockel R, Geldner G, Diefenbach C, Ulm K, Blobner M (2003). The use of neuromuscular monitoring in Germany [Article in German]. Anaesthesist.

[6] Appiah-Ankam J, Hunter JM (2004). Pharmacology of neuromuscular blocking drugs. Cont Educ Anaesth Crit Care Pain.

[7] McGrath CD, Hunter JM (2006). Monitoring of neuromuscular block. Cont Educ Anaesth Crit Care Pain.

[8] Adamus M, Belohlavek R, Koutna J, Vujcikova M, Janaskova E (2006). Cisatracurium vs. rocuronium: a prospective, comparative, randomized study in adult patients under total intravenous anaesthesia. Biomed Pap Med Fac Univ Palacky Olomouc Czech Repub.

[9] Baillard C, Gehan G, Reboul-Marty J, Larmignat P, Samama CM, Cupa M (2000). Residual curarization in the recovery room after vecuronium. Br J Anaesth.

[10] Xiaobo F, Jianjuan K, Yanlin W (2012). Comparison of the variability of the onset and recovery from neuromuscular blockade with cisatracurium versus rocuronium in elderly patients under total intravenous anesthesia. Braz J Med Biol Res.

[11] Tsai CC, Chung HS, Chen PL, Yu CM, Chen MS, Hong CL (2008). Postoperative residual curarization: clinical observation in the post-anesthesia care unit. Chang Gung Med J.

[12] JR Invernizzi, PD Gopalan (2016). Postoperative neuromuscular function following non-depolarising muscle blockade in patients at Inkosi Albert Luthuli Central Hospital, Durban. South Afr J Anaesth Analg.

[13] Viby-Mogensen J, Jørgensen BC, Ording H (1979). Residual curarization in the recovery room. Anesthesiology.

[14] Cammu G, de Baerdemaeker L, den Blauwen N, de Mey JC, Struys M, Mortier E (2002). Postoperative residual curarization with cisatracurium and rocuronium infusions. Eur J Anaesthesiol.

[15] Maybauer DM, Geldner G, Blobner M (2007). Incidence and duration of residual paralysis at the end of surgery after multiple administrations of cisatracurium and rocuronium. Anaesthesia.

